# A scoping review of researchers’ involvement in health policy dialogue in Africa

**DOI:** 10.1186/s13643-021-01745-y

**Published:** 2021-06-27

**Authors:** Doris Yimgang, Georges Danhoundo, Elizabeth Kusi-Appiah, Vijit Sunder, Sandra Campbell, Sanni Yaya

**Affiliations:** 1 University of Maryland Baltimore, Baltimore, USA; 2 World Health Organization, Geneva, Switzerland; 3 University of Alberta, Edmonton, Canada; 4 McMaster University, Hamilton, Canada; 5 School of International Development and Global Studies, University of Ottawa, 120 University Private, Ottawa, Ontario K1N 6 N5 Canada; 6 The George Institute for Global Health, Imperial College London, London, UK

**Keywords:** Health policy dialogue, Researchers’ involvement, Africa, Barriers, Facilitators

## Abstract

**Background:**

Improving evidence-informed policy dialogue to support the development and implementation of national health policies is vital, but there is limited evidence on researchers’ roles in policy dialogue processes in Africa. The objective of this study is to examine researchers’ involvement in health policy dialogue in Africa.

**Methods:**

The database search of this scoping review was conducted from inception to January 24, 2021, by an expert searcher/librarian to determine the extent of evidence, barriers, and facilitators of researchers’ involvement in health policy dialogues in Africa. PROSPERO, Wiley Cochrane Library, OVID Medline, OVID EMBASE, OVID PsycINFO, OVID Global Health, EBSCO CINAHL, BASE (Bielefeld Academic Search Engine), and Google/Google Scholar were searched using key words representing the concepts “policy dialogue”, “health”, and “Africa”. No limits were applied. A narrative summary of results was presented.

**Results:**

There were 26 eligible studies representing 21 African countries. Significant discrepancies in researchers’ involvement existed across countries. In 62% of the countries, there was suboptimal involvement of researchers in policy dialogues due to no or partial participation in policy dialogues. Major barriers included limited funding, lack of evidence in the public health field of interest, and skepticism of policymakers. The presence of an interface for exchange, demand for scientific evidence, and donors’ funding were the most reported facilitators.

**Conclusions:**

To improve the uptake of evidence in health policy-making processes, an environment of trust and communication between policymakers and researchers must be established. Policymakers need to demonstrate that they value research, by providing adequate funding, promoting knowledge translation activities, and supporting personal and professional development opportunities for researchers.

**Supplementary Information:**

The online version contains supplementary material available at 10.1186/s13643-021-01745-y.

## Background

The gap between research and health policy development in low- and middle-income countries appears uncontended. Mounting evidence demonstrates that the health policy-making process in these countries is prescriptive, insufficiently evidence-based, and inconsiderate of contexts [1, 2]. The political tussle over the formation of values and ideas informing health policy directives increases the propensity for misusing the already scarce resources, which hampers the economic growth and worsens the instability of social systems [3]. Globally, researchers and academic experts, largely considered to be well-informed citizens, discourage the formation of policies based on decision-makers’ personal ideas, interests, or experiences [1, 4]. Looking at the formation of health policies in the African context, Siron and his colleagues [5] identified that the increasingly politicized health policy decisions are driven by the ideological motivations of stakeholders who overlook research evidence to advance their political interests.

The global concern for evidenced-informed policies in Africa relates to the humanitarian call to build the capacity of local policy actors for developing evidence informed health policies [3, 6, 7]. For instance, in 2011 the European Union, World Health Organization (WHO), and the Government of Luxembourg formed a partnership to help build the capacity of developing countries for evidence-informed health policies using policy dialogue [6]. Nonetheless, researchers’ involvement in policy dialogue in Africa remains unclear.

The complicated relationship between researchers and politicians sparked the creation of deliberative dialogue platforms, to facilitate the process of multiple stakeholders participating in evidence-informed policy decision-making [7]. Policy dialogue is a participatory approach to policy making based on evidence, deliberative discussion, workshop interaction, and consultation [8, 9]. A strong and independent policy research organization can play an important role in informing and shaping policies for the greatest good of the public [10]. The sustained development of capacity for health policy research and uptake of evidence is a key priority for WHO. The Organization promotes efforts towards fostering and encouraging a culture of evidence-informed decision-making through strengthening the capacity of research institutions and stimulating the interest of policymakers [11].

The process of policy influence through research is complex and non-linear and demands much more than an ability to produce high caliber research [12]. Some researchers operate in environments with traditions of social participation; others have limited input. Despite these differences, all health researchers must grapple with political realities in their local contexts. A reflective and cross-sectional analysis of national deliberative policy dialogue workshops in six west African countries reported by Riddle and Dagenais [7] suggested that there are several challenges related to the facilitation of successful evidenced-informed policy dialogue. As such, researchers need the capacity not just to produce knowledge, but to navigate in complex terrain. There is scant evidence on factors that affect researchers’ involvement and contribution to health policy dialogue in Africa. This paper explores the current practice of policy dialogue in relation to the incorporation of research evidence for health policy formation in the African context. Therefore, a scoping review was conducted to examine the researchers’ involvement range, researchers’ roles, facilitators, and barriers to researcher’s involvement in health policy dialogue in Africa.

## Methods

A scoping review was conducted to determine the range and nature of research activity concerning the barriers and facilitators of researchers’ involvement in health policy dialogues in Africa. A scoping review is a research methodology used to identify key factors related to a concept, to map the available evidence, and discuss the concept or key factors [13]. The preferred reporting items for systematic reviews and meta-analyses extension for scoping reviews (PRISMA-ScR) checklist was used to present the study methodology and findings [14, 15].

### Identification of the research question

Our research question was “what are the facilitators and barriers to researcher’s involvement in health policy dialogue in Africa?”

### Protocol

We used the preferred reporting items for systematic reviews and meta-analysis protocols (PRISMA-P) to develop our protocol, which is available in Additional file 1.

### Identification of the relevant studies

A search was executed by an expert searcher/librarian (SC) in the following databases: PROSPERO, Wiley Cochrane Library, OVID Medline, OVID EMBASE, OVID PsycInfo, OVID Global Health, and EBSCO CINAHL, using controlled vocabulary (e.g. MeSH, Emtree, etc.) and key words representing the concepts “policy dialogue” and “health” and “Africa”. Databases were searched from inception to January 24, 2021. To broadly capture the existing literature, no limits were applied. Grey literature searches were conducted in BASE (Bielefeld Academic Search Engine) and Google/Google Scholar. Results (*n* = 513) were exported to RefWorks citation management system. Detailed search strategies are available in Additional file 2.

### Study selection

Two co-authors (DY and GD) independently screened titles and abstracts of identified papers. After full-text screening by a co-author (EK-A), papers were then categorized into case studies, commentary, structured reflection, and quantitative studies. Four co-authors (DY, EK-A, GD, VS) carried out full-text extraction using a data extraction sheet developed for the purpose of this study. Researchers first verified that papers met inclusion criteria and focused on the topic of interest. Discrepancies in reviewers’ responses at abstract and full article screenings were resolved through discussion.

### Inclusion and exclusion criteria

Studies were included in this review if they met the following criteria: (1) peer-reviewed papers or grey literature on health policy dialogue, (2) reporting findings from programs or interventions conducted in Africa, and (3) describing country-level policy dialogue. Studies were excluded if papers were reviews, protocols, editorials, or other opinion pieces, and authors were not identified.

### Data charting

Each paper was independently reviewed by two authors, who discussed charted data and updated the data extraction sheet accordingly. Any disagreements were resolved through further adjudication by a third author. An excel data sheet was used to organize data extracted from each paper into themes. Information extracted from the selected studies were organized and categorized as follows: authors and publication date, country, study type, public health issue that triggered the policy dialogue, description of the policy window, organizers of the policy dialogue, actors involved in the policy dialogue and their roles, contribution of researchers to the policy dialogue, presence of local researchers, barriers and facilitators of researchers’ involvement in policy dialogue, and outcome of the policy dialogue.

### Collating and summarizing findings

A thematic data-synthesis was performed to identify contextual barriers and facilitators of researcher’s involvement in health policy dialogue. The synthesis includes useful information on the underlying processes of the public health issue that triggered the policy dialogue, policy window under scrutiny, actors involved in the policy dialogue and their roles, and contribution of researchers to the policy dialogue.

To provide an overview of the findings, a narrative summary of results is presented given the heterogeneity of study designs, objectives, and outcomes.

## Results

### Search results

**Fig. 1:**
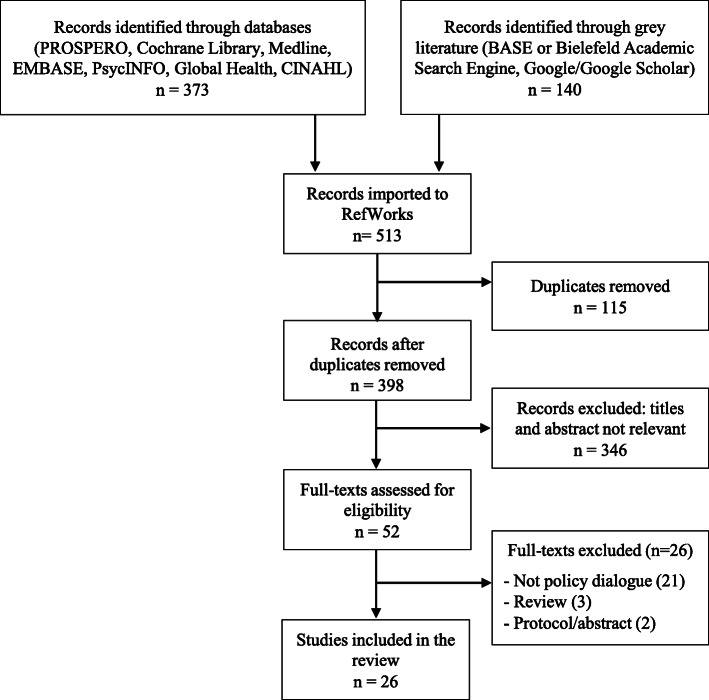
Study flow diagram

A preliminary search of scientific databases and grey literature yielded a total of 513 studies. After removing the duplicates (*n* = 115), titles and abstracts of 398 studies were screened excluding an additional 346 studies and leaving a sample of 52 studies. Screening of full texts yielded a total sample of 26 studies eligible for this review. A complete study flow diagram is shown in Fig. 1.

### Study characteristics

Studies included in this review were published between 2005 and 2020. Studies focused on Africa with twenty-one [16] countries represented and seven [7] multinational studies, involving more than one African country. Uganda had the most studies (*n* = 6), followed by South Africa (*n* = 3), Benin (*n* = 3), Nigeria (*n* = 3), Burkina Faso (*n* = 2), Ghana (*n* = 2), Malawi (*n* = 2), Mali (*n* = 2), Senegal (*n* = 2), and Tanzania (*n* = 2). One study from each of the following countries was included: Cabo Verde, Cameroon, Chad, Guinea, Ivory Coast, Liberia, Morocco, Mozambique, Niger, Zambia, and Zimbabwe.

The most common study design was case study (*n* = 16) with qualitative data describing policy dialogue in specific settings. Additional qualitative studies included: commentary (*n* = 1), exploratory study (*n* = 1), desk review (*n* = 1), reflective analysis (*n* = 1), and participatory action research (*n* = 1). These studies were primarily supported by international funding (19 out of 26), and 21 studies receiving technical support from international agencies or governments, with the main funder being WHO (*n* = 16). Local government officials (such as the President or Prime Minister) or ministry of health (MoH) initiated the dialogue in 11 studies. Content areas varied across studies with the most predominant being malaria (*n* = 5), HIV/AIDS (*n* = 4), maternal and child health (*n* = 5), and discussion of gaps in evidence uptake in health policy (*n* = 4).

### Stakeholders participating in policy dialogue

**Fig. 2:**
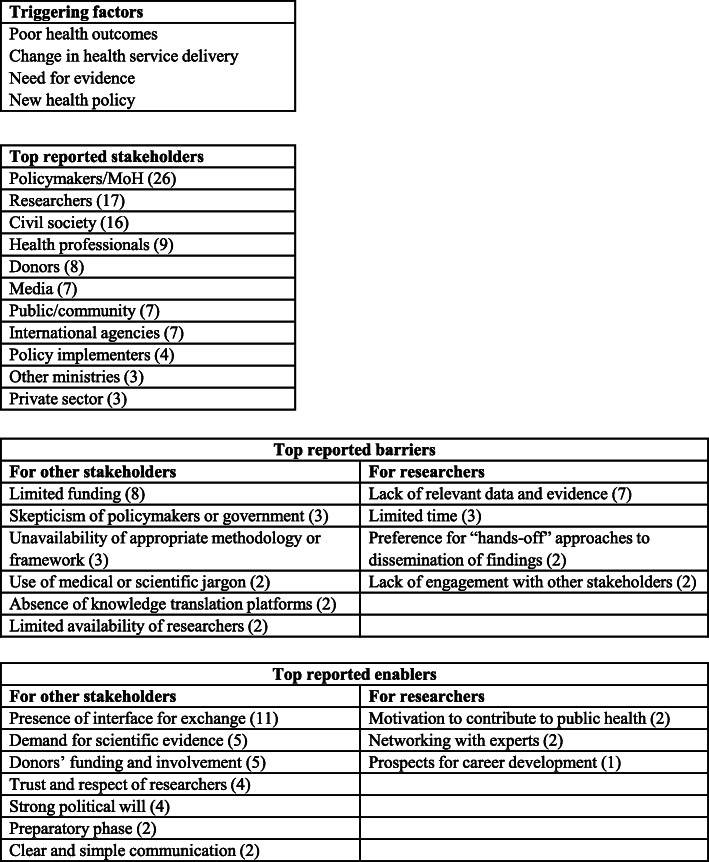
Summary of triggering factors, barriers, and facilitators of researchers’ involvement in policy dialogue

Policy dialogues were mainly organized in the context of poor health outcomes, change in health service delivery, the need for research evidence in policy development, or the development of new health policies (Fig. 2). Varied stakeholders attended policy dialogue with the participation of researchers acknowledged in 18 studies, in which local researchers were involved as subject matter experts in the public health being discussed (Table 1). In 65% of the studies (*n* = 17), researchers actively participated in the policy dialogue, and they performed tasks such as generating evidence, developing policy briefs, organizing or facilitating policy dialogues, assisting policymakers by summarizing evidence and making recommendations, and supporting the policy implementation process. Researchers had a more passive role in one study [37]; their responsibilities consisted of organizing the policy dialogue and collecting data without being part of the discussion. Policymakers attended almost all the meetings (25 out of 26). Only one study did not report the participation of policymakers in the policy dialogue due to the lack of government initiative to change policy regarding HIV/AIDS health service delivery in South Africa [33]. Civil society organizations initiated the policy process, established platforms to discuss with other stakeholders (including researchers, business organizations, and the African trade union), and collaborated on the development of policy plans [33]. After much advocacy efforts that resulted in antiretroviral price reductions, the government joined in the efforts to improve HIV/AIDS health service delivery [33]. Other commonly reported stakeholders attending policy dialogues included civil society (*n* = 16), health professionals (*n* = 9), donors (*n* = 8), the media (*n* = 7), the public or community members (*n* = 7), and international agencies (*n* = 7).

**Table 1:** Characteristics of publications on policy dialogue in Africa

Author and date	Country	Study type	Public health issue	Participants in policy dialogue	Role of researchers	Presence of local researchers
Ade et al. 2016 [17]	Guinea	Case study	National health policy	MoH, civil society, development partners, Ministry of Environment	Not reported	Not reported
Akhnif et al. 2020 [18]	Morocco	Case study	Health financing	Key ministries, media, parliamentarians, private sector, researchers, civil society, health professionals, technical and financial partners	Active—organized workshops, participated and contributed to dialogue, and documented discussions	Yes
Berman et al. 2015 [16]	Malawi	Commentary	Development of knowledge translation platform	Researchers, policymakers, implementers, civil society	Active—generated evidence, developed policy briefs, facilitated policy dialogue	Yes
Burris et al. 2011 [19]	Ghana	Case study	HIV-herpes simplex virus type-2 interaction	Researchers, policymakers,	Active—generated evidence, contributed to policy development	Yes
De Carvalho et al. 2014 [20]	Ghana	Case study	Aging and health	Key ministries, the Ghana Health Service, teaching hospitals, professional bodies, HelpAge Ghana, WHO	Not reported	Not reported
Dossou et al. 2018 [21]	Benin	Case study	User fees for caesarian section	MoH, implementers, healthcare professionals, economists, civil society	Not reported	Not reported
Dovlo et al. 2016 [6]	Multinational—Cabo Verde, Chad, Mali	Exploratory study	Improvement of national health development	MoH, donor agencies, civil society	Not reported	Not reported
Johnson et al. 2020 [22]	Nigeria	Case study	Maternal child health	Policymakers, technical and financial partners, civil society, researchers, healthcare professionals	Active—participated in discussions,	Yes
Kinoti et al. 2014 [23]	Multinational—Malawi, Uganda, Zambia	Not reported	Abortion complications	Researchers, policymakers, healthcare providers	Active—conducted research, disseminated findings, participated in dialogues, developed action plans	Yes
Kirigia et al. 2016 [24]	Multinational—African region	Not reported	Increase uptake of evidence in health policy and practice	Researchers, policymakers, ministries, WHO, public	Active—presented findings, led discussions	Yes
Mbonye et al. 2013 [25]	Uganda	Not reported	Malaria, infectious and communicable diseases	Researchers, policymakers, civil society, media	Active—developed and reviewed policy briefs, participated in policy workshops	Yes
Mc Sween-Cadieux et al. 2018 [26]	Burkina Faso	Mixed methods	Road traffic injuries	Researchers, health professionals, civil society, police, government	Active—conducted research, organized policy workshop	Yes
Mubyazi et al. 2005 [27]	Tanzania	Case study	Antimalarial drug policy	Researchers, policymakers, drug manufacturers, media, practitioners, public	Active—generated evidence, disseminated findings, participated in discussions	Yes
Nabyonga-Orem et al. 2014 [28]	Uganda	Case study	Malaria treatment policy change	Researchers, policymakers, MoH, donors, parliamentarians, civil society, media, communities	Active—generated evidence, participated in policy development	Yes
Nabyonga-Orem et al. 2016 [29]	Liberia	Case study	Policy dialogue before and after the Ebola outbreak	Policymakers, donors, NGO, policy implementers, MoH	Not reported	Not reported
Odoch et al. 2015 [30]	Uganda	Desk review	Male circumcision for HIV prevention	Researchers, MoH, donors, media, civil society, public	Active—generated evidence, participated in policy negotiation, formulation, communication, and implementation	Yes
Ongolo-Zogo et al. 2014 [31]	Multinational—Cameroon and Uganda	Case study	“Evidence to policy” around priority topics	Researchers, policymakers, international bureaucrats, knowledge brokers, civil society, media	Active—generated evidence, prepared policy briefs, organized dialogues	Yes
Paul et al. 2020 [32]	Multinational—Benin and Senegal	Case study	Universal health coverage	Policymakers, health professionals, public	Not reported	Not reported
Ridde et al. 2017 [7]	Multinational—Benin, Burkina Faso, Ivory Coast, Mali, Niger, Senegal	Reflective and cross-sectional analysis	New health policies on health coverage and employment	High-level decision makers	Not reported	Not reported
Sabi et al. 2017 [33]	South Africa	Case study	Improvement of HIV/AIDS health service delivery	Researchers, civil society, business organizations, African trade union	Active—developed policy proposals	Yes
Ssengooba et al. 2011 [34]	Uganda	Case study	Prevention of mother-to-child transmission and safe male circumcision	Researchers, policymakers, media, donors, public	Active—participated in policy formulation and implementation, secured funding for programs	Yes
Uneke et al. 2015 [35]	Nigeria	Cross-sectional analysis	Strategies to control infectious diseases of poverty (malaria, schistosomiasis, and lymphatic filariasis)	Researchers, policymakers, MoH, civil society, health professionals	Active—provided support and mentorship to policymakers for policy development, participated in policy dialogue	Yes
Wammanda et al. 2020 [36]	Nigeria	Case study	Serious bacterial infection in young infants	MoH, WHO, civil society, policymakers, program implementers, health professionals	Not reported	Not reported
Webber et al. 2018 [37]	Tanzania	Participatory action research	Maternal health	Policymakers, village leaders, community members	Passive—organized participatory action research and collected data	Yes
Woelk et al. 2009 [38]	Multinational—Mozambique, South Africa, Zimbabwe	Case study	Use of magnesium sulphate in the treatment of eclampsia in pregnancy; use of insecticide treated bed nets and indoor residual household spraying for malaria vector control	Researchers, policymakers, MoH, civil society, international agencies	Active—generated evidence, contributed to policy development and review, collaborated with health officials, chaired policy-making committee	Yes
Young et al. 2018 [39]	South Africa	Case study	Use of research evidence in policy	Policymakers and research buddies	Active—partnered with policymakers and provided scientific support	Yes

### Researchers’ roles in health policy dialogue across countries

Few countries featured successful researchers’ involvement in all their studies or national policy dialogues. For instance, researchers actively participated in all six studies from Uganda. Researchers helped generate evidence (*n* = 4), developed and reviewed policy briefs (*n* = 6), and organized dialogues or participated in discussions (*n* = 6). Major facilitators in this setting included shared platforms for knowledge translation, sustained collaboration between researchers and policymakers, and demand for research evidence. In South Africa, researchers participated in all three studies, but their roles varied based on their involvement. Sabi and colleagues described the involvement of researchers in developing policy proposals to support change in HIV/AIDS health delivery service initiated by civil society organizations [33]. Young and colleagues reported that researchers were paired with policymakers to increase the uptake of evidence in health policy decisions; researchers helped with clarifying research questions, appraising systematic reviews, preparing short evidence summaries, and providing feedback to policymakers [39]. In South Africa, Mozambique, and Zimbabwe, researchers participated in shaping policies around eclampsia treatment and malaria control, and they participated in generating evidence and policy development in collaboration with other stakeholders [38]. Facilitators of researchers’ involvement in policy dialogues from these three countries included the involvement of local researchers in randomized trials conducted to generate evidence to support policy change. In Malawi, researchers actively participated in all two policy dialogues: one addressing abortion complications [23], and the other targeting the development of a knowledge translation platform [16]. To promote evidence uptake in health policy dialogue through the development of a shared platform, the Ministry of Health built partnerships with different stakeholders and defined roles of each actor in the process of making evidence-informed health policies [16]. Researchers actively participated in workshop’s facilitation and building capacity in developing research summaries and policy briefs [16].

Several countries had researchers engaged in some of their policy dialogues but not others. For instance, one of the two studies conducted in Burkina Faso reported researchers’ participation in the policy dialogue. Researchers conducted the research to generate evidence around road traffic injuries, and they organized policy workshops to disseminate findings and discuss recommendations with other stakeholders [26]. Researchers had an active role in one of the two studies from Tanzania [27], while the other study reported a passive role [37]. Researchers summarized and disseminated evidence, and they developed a research policy brief to highlight antimalarial drug resistance and the need for a policy change; stakeholder workshops were organized by the ministry of health and WHO to discuss evidence and recommendations for policy change [27]. A key facilitator of researchers’ involvement in Tanzania included the need for clear communication of research findings. One of the two studies conducted in Ghana reported researchers’ involvement in the policy dialogue. Researchers played a central role in trials to generate evidence and develop policy briefs [19]. The other study used WHO technical assistance to appraise evidence and draft policy briefs discussed during policy dialogue with national stakeholders [20]. A key limitation in Ghana was that donors’ interests took the priority in the policy agenda. Two of the three studies from Nigeria reported researchers’ engagement in the policy dialogue process [22, 35]. Uneke and colleagues described a mentorship program that was organized to build capacity of policymakers in developing policy briefs; policymakers drafted policy briefs on the control of infectious diseases of poverty, with technical support and mentorship of researchers [35]. These policy briefs were further evaluated and discussed during a policy dialogue, in which key stakeholders, including researchers, participated [35]. Johnson and colleagues reported on the Nigeria Research Days that were organized to discuss policies on maternal and child health by allowing a dialogue among various stakeholders, including researchers and policymakers [22]. Researchers were engaged in the preliminary phase to prepare for the policy dialogue, presented findings, and participated in discussions during the dialogue [22]. The last study from Nigeria briefly described a policy dialogue organized by the Federal MoH with the support from WHO to discuss the adoption of the WHO possible serious bacterial infection guideline [36].

Researchers were engaged in the policy dialogue process in Cameroon, Morocco, and Zambia, although there was only one study per country. Morocco, the sole Northern African country included in this review, organized a policy dialogue to discuss health financing [18]. Researchers were involved in synthesizing existing evidence, organizing the policy dialogue, and contributing to the development of national recommendations for a health financing strategy. Facilitators of researchers’ engagement included external funding, a preliminary phase to prepare the policy dialogue, and conceptualization of the dialogue to achieve the desired goals (e.g. selection of topics and subject matter experts to facilitate discussions) [18]. In Cameroon, the study described Evidence Informed Policy Network (EVIPNet) and reported researchers’ involvement in the development of policy briefs and participation in policy dialogue [31]. Between 2008 and 2012, 12 evidence briefs were produced, and seven policy dialogues were organized; facilitators of successful researchers’ involvement reported in this study included the support of external funding and a formal knowledge translation platform, which was comprised of researchers as staff [31]. A multinational study including Zambia reported active participation of researchers throughout the policy-making process [23].

Local researchers were involved in all 18 policy dialogues that reported the participation of researchers. However, studies reviewed did not provide enough information to determine whether local and external researchers had similar or different contributions to policy dialogues.

Studies in multiple Western and Central African countries (Benin, Cabo Verde, Chad, Guinea, Ivory Coast, Liberia, Mali, Niger, and Senegal) did not report researchers’ involvement in policy dialogues. In Guinea, researchers did not participate in the policy dialogue, but they evaluated its outcomes [17].

### Barriers to researchers’ involvement in policy dialogue

The most common barrier reported was limited funding to support policy dialogue activities (*n* = 8), which include research for evidence generation and stipends or reimbursements for incidental costs to stakeholders attending policy dialogues. Lack of funding influenced the ability to support an appropriate number of participants in the policy dialogue [25] and was reported as an obstacle to sustainability [16]. Reliance on donor funding resulted in donor interests being the priority of the policy agenda [19]. There was also skepticism around research funded by donors because of the possibility of conflict of interest [28]. However, high-level decision makers in the government sometimes expressed doubt about research evidence because of their personal beliefs, cultural values, and concerns regarding the impact of new interventions. For instance, in Uganda, the safe male circumcision policy process was delayed due to opposition from high-level political leaders, who were concerned about feasibility of the intervention and the unintended harmful impact of such a policy on the community as the public might misinterpret the intent of such interventions [34].

Another barrier reported was the absence of knowledge translation platforms (*n* = 2) resulting in decreased communication and dialogue between researchers and policymakers (*n* = 2). Poor collaboration between researchers and stakeholders in health policy (policymakers, civil society, media, industry, and public) hampers learning and translation of research findings, which widens the trust gaps and hinders the development of innovative and effective public health interventions [24].

For researchers, the most reported barrier was lack of relevant data and evidence to inform the policy dialogue (*n* = 8), and few studies reported that research evidence was not relevant to local contexts, highlighting the need for local evidence to support the policy process (*n* = 4). Researchers were more likely to pursue rewarding academic interests that were sometimes disconnected to the needs of the community or policy priorities (*n* = 2). As a result, few studies reported limited availability of researchers in the field of interest (*n* = 3). On the other hand, researchers perceived active involvement in the policy process as time consuming and demanding (*n* = 3). Instead of being actively engaged in the policy process (dissemination of findings and dialogue with policymakers and other stakeholders), some researchers preferred a “hands off” approach like sharing reports or publications with other stakeholders with no desire for further involvement (*n* = 2). In fact, building trust and relationships between policymakers and researchers requires time and commitment from both parties [39].

Among the studies that did not engage researchers in the policy dialogue (*n* = 8) reported barriers included limited funding (*n* = 3), poor methodology and coordination of policy dialogue (*n* = 2), and poor data quality or lack of evidence on the public health issue of interest (*n* = 2). Policymakers’ limited skills to find and evaluate research evidence led to low uptake of evidence, which created delays in decision-making [6]. A study that evaluated policy dialogues in Benin and Senegal did not acknowledge the participation of researchers as being trivial in policy dialogue processes [32].

### Facilitators of researchers’ involvement in policy dialogue

Out of the 18 studies that reported facilitators of researchers’ involvement in policy dialogue, the majority highlighted the need for a knowledge translation platform, a shared platform for exchange and decision-making (*n* = 11). This platform may take the form of a research network [38], institutional collaboration [25], regional cooperation [23], forum [29], dissemination workshop [26], or support network like ‘research buddies’ [39]. Restoration of trust between researchers and policymakers, respect for researchers’ objectivity, and careful selection of prominent researchers to facilitate dialogues were reported facilitators of this process (*n* = 4). The demand for scientific evidence (*n* = 5) coupled with simple and clear communication from researchers (*n* = 2) were also major facilitators of researchers’ involvement. The availability of funding from donors and their involvement were reported as enabling factors (*n* = 5). Strong political will (*n* = 4) and preliminary discussions in preparation for the policy dialogue (*n* = 2) were also identified as major facilitators of successful engagement of relevant stakeholders. A preparatory phase helps to better organize the policy dialogue process by identifying the needs of stakeholders before the actual dialogue and meeting their expectations [18, 22].

For researchers, incentives for their involvement in policy dialogue included the following: motivation to contribute to public health, leading to advocacy for specific causes (*n* = 2); networking with experts in the field of interest (*n* = 2); and prospects for career development (*n* = 1).

## Discussion

This scoping review aimed to analyse the barriers and facilitators of researchers’ involvement in health policy dialogue in Africa. This study found that internal factors (related to researchers) that motivated researchers include advocacy, personal, and professional development. The main factor hampering their involvement in policy dialogue was the absence of a conducive environment, which include financial resources to focus their research on relevant public health issues and to get involved in policy-making activities. External factors (related to the political environment) that enabled researchers’ involvement in policy dialogue include the following: an appreciation for researchers’ contribution, the presence of a knowledge translation platform, funding, and clear communication between researchers and policymakers. Findings of this scoping review are consistent with other research. Several research studies revealed numerous factors that determine whether and how research evidence is taken into account in decision-making: decision-makers’ opinions about research utility, their skill in interpreting and using research evidence, and whether there is a supportive context for its use [2, 40]. Likewise, Moat and colleagues show that decision-makers are influenced by institutional constraints, interest group pressures, personal convictions and values, external factors such as economic recession or elections, external funding, and research data [12]. It is worth noting that in many African countries, researchers’ involvement in health policy dialogue remains sub-optimal. Thirty-three percent of the countries (*n* = 9) represented in this study did not report the participation of researchers in the policy process. In an additional 15% (*n* = 4) of the countries, researchers were involved in only half of the health policy dialogues. Major issues reported in such instances are lack of evidence on public health issues discussed and poor methodology during the policy dialogue. The absence of research evidence during the policy dialogue generally results in the use of anecdotal evidence [20], which hardly yield effective interventions. Participation of researchers in the policy process has also been reported to be beneficial to policymakers because capacity-building activities could be developed to improve their skills regarding evidence-based policy-making processes [16]. This finding highlights the need to develop knowledge translation platforms that encourage the involvement of all key stakeholders in the entire process of health policy-making.

In most African countries, international donors can have an influence on policy processes with a bearing on the proposed solutions [41]. It has been reported that external donors impose their preferences rather than discussing several options for addressing a locally identified problem [42]. However, country ownership and donors’ influence has successfully coexisted in several contexts [43]. In this study, local researchers were involved in policy dialogues even when funding originated from international organizations or partnerships. Although the studies reviewed do not provide a distinction between the roles of local and external researchers, the involvement of local researchers was reported where researchers contributed to policy dialogues. Previous research has emphasized the importance of engaging local researchers on public health issues relevant to their countries and expertise. Local researchers have pivotal roles to play, first and foremost by assisting the MoH with key studies and localized and decentralized information. They also have an important advocacy role, i.e. bringing to attention priority health issues and offering options to solve them [9]. In practice, however, stakeholders who provide funding may be perceived as more important than others. A tacit establishment of a certain hierarchy of stakeholders can affect local researchers’ abilities to influence health policy dialogues.

In many African countries, the production of research evidence is often very limited [44]. Due to limited funding in the research sector, researchers are highly involved in consultancy tasks. These consultancy contracts require commissioned reports and researchers may not have enough time to increase their ability to apply scientific reflection [45]. Furthermore, evidence shows that researchers’ influence on policy is shaped by their reputations as independent researchers that provide credible research and their agility in navigating the local policy landscape and participating in policy debates [45]. Independence is closely linked to financial sustainability. Core funding can help position grantees for policy influence by increasing their independence and credibility, staff reputations, and communication skills. Sustaining this independence over the long-term demands strengthening internal capacities [11].

Engaging policymakers early in the research cycle helps to ensure uptake of evidence. Researchers’ ability to influence policies is strongly shaped by external factors, especially political barriers. Their agility in responding to these shifts rests in part on their skills in engaging with stakeholders, so that they are attuned to the environment. Choosing the right points of entry for policy engagement is equally important [46]. WHO stresses the value of closer collaboration between research organizations and the policymakers they seek to influence, so that evidence creation is better aligned with policy priorities [11]. At the same time, researchers need to maintain an ethical and impartial stance, ensuring that multiple perspectives inform their research. Achieving this balance demands considerable skill. Achieving policy influence takes a “whole organization” approach. Strong research institutions alone are insufficient to create a culture of evidence-based policy-making [12]. Citizens must be able to demand accountability and participate in decision-making [47]. It is important that research institutions involve community representatives, the media, and advocates for marginalized groups directly in research, from project proposal to completion stages. This strengthens research design while helping communities understand the value of evidence and their own participation in the policy-making process [10]. Donors can help position researchers for influence through flexible funding arrangements that provide for organizational strengthening, while reinforcing researchers’ independence.

To properly evaluate the effectiveness of policy dialogues in Africa, more studies describing policy-making processes around various public health issues need to be published. Out of the 21 countries included in our review, only four countries (15%) had at least three studies. As more studies per country are being published, a more complete picture of country-level policy landscapes could be presented, and country-specific tailored interventions could be implemented to develop productive and consultative platforms where health policies could be discussed.

## Strengths and limitations

The major strength of this scoping review is the focus on researchers’ involvement in health policy dialogues in Africa. Many studies have been published on policy-making processes in various African countries, but there is limited evidence on barriers and facilitators of researchers’ involvement in this process. The main limitation of this study is the limited amount of information on the policy-making process available at the country-level as most countries represented in this scoping review had only one publication (*n* = 11) and were sometimes published as part of multinational studies (*n* = 8).

## Conclusion

This review provides an overview of evidence on researchers’ involvement in health policy dialogue in Africa and highlights barriers and facilitators of this involvement. The most important factor related to researchers’ involvement was the presence of a conducive environment that would support and value research while promoting knowledge translation activities. Such an environment would only be functional with adequate funding, trust and communication between policymakers and researchers, and promising personal and professional development opportunities for researchers. Discrepancies exist across countries and sometimes within a specific country. More than half of the countries represented had partial to no researchers’ involvement in policy dialogues. While low- and middle-income countries are still striving to increase the uptake of evidence in health policies, these findings highlight areas for improvement. Further evidence on policy processes within specific settings are needed to better inform interventions or practices.

## Supplementary Information

**Additional file 1.** Study protocol.

**Additional file 2.** Description of search strategies in various databases and grey literature. Searches conducted in January 2021.

## Data Availability

No additional data available.

## Abbreviations

MoH: Ministry of Health
WHO: World Health Organization
