# Supplementary material for: A scoping review of researchers’ involvement in health policy dialogue in Africa
Source: Syst Rev. 2021 Jun 27;10:190. doi: 10.1186/s13643-021-01745-y (PMC8236190; doi:10.1186/s13643-021-01745-y)
Supplement: Supplementary file 1 — Additional file 1. Study protocol. [file 13643_2021_1745_MOESM1_ESM.docx]

**STUDY PROTOCOL**

**A scoping review of researchers’ involvement in health policy dialogue in Africa**

**Authors**

**Doris Yimgang** ([doris_yimgang@umaryland.edu](mailto:doris_yimgang@umaryland.edu)) is affiliated with the University of Maryland Baltimore. She developed the protocol, screened papers, drafted sections of the manuscript, and critically reviewed the final manuscript.

**Georges Danhoundo** ([danhoundog@who.int](mailto:danhoundog@who.int)) is affiliated with the World Health Organization. He conceived the study, developed the protocol, screened papers, drafted the manuscript, supervised the study, and critically reviewed the final manuscript.

**Elizabeth Kusi-Appiah** ([kusiappi@ualberta.ca](mailto:kusiappi@ualberta.ca)) is affiliated with the University of Alberta. She developed the protocol, screened papers, and critically reviewed the final manuscript.

**Vijit Sunders (**[vijit.sunder@gmail.com](mailto:vijit.sunder@gmail.com)) is affiliated with McMaster University. He developed the protocol, screened papers, and critically reviewed the final manuscript.

**Sandra Campbell (**[scampbel@ualberta.ca](mailto:scampbel@ualberta.ca)) is affiliated with the University of Alberta. She performed literature searches and reviewed the final manuscript.

**Sanni Yaya** ([hsanniya@uottawa.ca](mailto:hsanniya@uottawa.ca)) is affiliated with the University of Ottawa and the corresponding author. He supervised the study, provided guidance, and critically reviewed the manuscript.

**Corresponding author**

Sanni Yaya, PhD

School of International Development and Global Studies

University of Ottawa

120 University Private

Ottawa, Ontario K1N 6N5

[sanni.yaya@uottawa.ca](mailto:sanni.yaya@uottawa.ca)

**Funding:** None.

**INTRODUCTION**

A new wave of scholarship in policy studies has stressed the role of dialogue in policy development. This approach to policy development contributes important insights to policy analysis by showing how policy dialogue can shape policy outcomes (Dovlo et al. 2016; Rajan et al. 2015).

Addressing policy dialogue in Africa is particularly important because research on policy implementation in Africa is particularly limited. According to Saetren (2005), only 4% of the research on policy implementation worldwide has been conducted in Africa. There is scant evidence on both health policy dialogue processes and outcomes in Africa.

Examining the health policy dialogue processes and outcomes in Africa will provide insight into the factors leading to successful or unsuccessful engagement of researchers in the policy-making processes and implementation, but there is limited evidence on researchers’ roles in policy dialogue processes in Africa. The objective of this study is to examine researchers’ involvement in health policy dialogue in Africa.

**METHODS**

| **Table 1. Inclusion and exclusion criteria** | |
| --- | --- |
| **Inclusion criteria** | **Exclusion criteria** |
| Africa | Areas outside of Africa |
| Peer-reviewed articles on health policy dialogue | Publications that do not report specifically on health policy dialogue |
| Grey literature on health policy dialogue | Editorials and International dialogue on health policy |
| Country level policy dialogue | Anonymous authors |

**Search strategy**

A search will be executed by an expert searcher/librarian (from inception to January 2021) on the following databases:

- PROSPERO
- Wiley Cochrane Library
- OVID Medline
- OVID EMBASE
- OVID Global Health
- EBSCO CINAHL

Using controlled vocabulary (eg:  MeSH, Emtree, etc) and key words representing the concepts “policy dialogue" and “health”, and “Africa”.   No limits will be applied. Grey literature searches will be conducted in:

- BASE (Bielefeld Academic Search Engine)
- Google/Google Scholar

Results will be exported to RefWorks citation management system.

**Data extraction**

At least two authors will independently screened titles, abstracts, and full texts of all the identified papers. Articles that do not meet inclusion criteria will be excluded.

Authors will develop a data extraction sheet for this study to describe the variables that need to be abstracted from the papers. Variables of interest include:

- Public health issue that triggered the policy dialogue
- Organizers of the policy dialogue
- Actors involved in the policy dialogue and their roles
- Contribution of researchers to the policy dialogue
- Presence of local researchers
- Barriers and facilitators of researchers’ involvement in policy dialogue
- Outcome of the policy dialogue.

**Data synthesis**

A thematic data-synthesis will be performed to identify contextual barriers and facilitators to researcher’s involvement in health policy dialogue.

A narrative summary of results will be presented to provide an overview of the findings.
