# Supplementary material for: A scoping review of researchers’ involvement in health policy dialogue in Africa
Source: Syst Rev. 2021 Jun 27;10:190. doi: 10.1186/s13643-021-01745-y (PMC8236190; doi:10.1186/s13643-021-01745-y)
Supplement: Supplementary file 2 — Additional file 2. Description of search strategies in various databases and grey literature. Searches conducted in January 2021. [file 13643_2021_1745_MOESM2_ESM.docx]

**Search strategies**

**Ovid MEDLINE(R) ALL <1946 to January 22, 2021>**

| **#** | **Search Statement** | **Results** |
| --- | --- | --- |
| 1 | africa*.mp. or exp AFRICA/ or (Algeria* or Angola* or Benin* or Botswana* or Burkina Faso or Burundi* or Cameroon* or Cape Verd* or Central African Republic or Chad or Tchad or Comoros or Congo or Congolese or Djibouti* or Egypt* or Equitorial Guinea or Eritrea* or Ethiopia* or Gabon or Gabonese or Gambia or Gambian or Ghana* or (Guinea not (fowl or pig*)) or Guinea Bissau or Ivory Coast or Kenya* or Lesotho or Liberia* or Libya* or Madagascar or Malawi* or Mali or Mauritania* or Mauritius or Morocco or Moroccan or Mozambique or Namibia* or Niger or Nigeria or Rwanda& or Sao Tome or Senegal* or Seychells or Sierra leone or Somalia* or Sough Africa* or Sudan* or Swaziland or Tanzania* or Togo or Tunisia* or Uganda* or Zambia* or Zimbabw*).ti,ab. | 553328 |
| 2 | (policymaking or policy making).mp. [mp=title, abstract, original title, name of substance word, subject heading word, floating sub-heading word, keyword heading word, organism supplementary concept word, protocol supplementary concept word, rare disease supplementary concept word, unique identifier, synonyms] | 21979 |
| 3 | management/ and (policy or policies).mp. | 0 |
| 4 | 2 or 3 | 21979 |
| 5 | 4 and (dialog* or discuss* or negotiat* or conversation*).ti. | 150 |
| 6 | health care policy/ and (dialog* or discuss* or negotiat* or conversation*).ti. | 0 |
| 7 | (((policy or policies) adj (dialog* or discuss or negotiat* or conversation*)) and health).mp. [mp=title, abstract, original title, name of substance word, subject heading word, floating sub-heading word, keyword heading word, organism supplementary concept word, protocol supplementary concept word, rare disease supplementary concept word, unique identifier, synonyms] | 192 |
| 8 | ((deliberative adj (dialog* or discuss or negotiat* or conversation*)) and health polic*).mp. | 17 |
| 9 | 5 or 6 or 7 or 8 | 332 |
| 10 | 1 and 9 | 78 |
| 11 | remove duplicates from 10 | 76 |

**Embase <1974 to 2021 January 21>**

| **#** | **Search Statement** | **Results** |
| --- | --- | --- |
| 1 | africa*.mp. or exp AFRICA/ or (Algeria* or Angola* or Benin* or Botswana* or Burkina Faso or Burundi* or Cameroon* or Cape Verd* or Central African Republic or Chad or Tchad or Comoros or Congo or Congolese or Djibouti* or Egypt* or Equitorial Guinea or Eritrea* or Ethiopia* or Gabon or Gabonese or Gambia or Gambian or Ghana* or (Guinea not (fowl or pig*)) or Guinea Bissau or Ivory Coast or Kenya* or Lesotho or Liberia* or Libya* or Madagascar or Malawi* or Mali or Mauritania* or Mauritius or Morocco or Moroccan or Mozambique or Namibia* or Niger or Nigeria or Rwanda& or Sao Tome or Senegal* or Seychells or Sierra leone or Somalia* or Sough Africa* or Sudan* or Swaziland or Tanzania* or Togo or Tunisia* or Uganda* or Zambia* or Zimbabw*).ti,ab. | 632433 |
| 2 | (policymaking or policy making).mp. [mp=title, abstract, heading word, drug trade name, original title, device manufacturer, drug manufacturer, device trade name, keyword, floating subheading word, candidate term word] | 7949 |
| 3 | management/ and (policy or policies).mp. | 9824 |
| 4 | 2 or 3 | 15917 |
| 5 | 4 and (dialog* or discuss* or negotiat* or conversation*).ti. | 97 |
| 6 | health care policy/ and (dialog* or discuss* or negotiat* or conversation*).ti. | 1055 |
| 7 | (((policy or policies) adj (dialog* or discuss or negotiat* or conversation*)) and health).mp. [mp=title, abstract, heading word, drug trade name, original title, device manufacturer, drug manufacturer, device trade name, keyword, floating subheading word, candidate term word] | 213 |
| 8 | ((deliberative adj (dialog* or discuss or negotiat* or conversation*)) and health polic*).mp. | 8 |
| 9 | 5 or 6 or 7 or 8 | 1286 |
| 10 | 1 and 9 | 104 |
| 11 | remove duplicates from 10 | 103 |

**APA PsycInfo <1806 to January Week 3 2021>**

| **#** | **Search Statement** | **Results** |
| --- | --- | --- |
| 1 | (africa* not "african american").mp. or exp AFRICA/ or (Algeria* or Angola* or Benin* or Botswana* or Burkina Faso or Burundi* or Cameroon* or Cape Verd* or Central African Republic or Chad or Tchad or Comoros or Congo or Congolese or Djibouti* or Egypt* or Equitorial Guinea or Eritrea* or Ethiopia* or Gabon or Gabonese or Gambia or Gambian or Ghana* or (Guinea not (fowl or pig*)) or Guinea Bissau or Ivory Coast or Kenya* or Lesotho or Liberia* or Libya* or Madagascar or Malawi* or Mali or Mauritania* or Mauritius or Morocco or Moroccan or Mozambique or Namibia* or Niger or Nigeria or Rwanda& or Sao Tome or Senegal* or Seychells or Sierra leone or Somalia* or Sough Africa* or Sudan* or Swaziland or Tanzania* or Togo or Tunisia* or Uganda* or Zambia* or Zimbabw*).ti,ab. | 76927 |
| 2 | ((policymaking or policy making).mp. or Policy Making/) and (dialog* or discuss* or negotiat* or conversation*).ti. | 321 |
| 3 | Health Policy/ and (dialog* or discuss* or negotiat* or conversation*).ti. | 0 |
| 4 | (((policy or policies) adj (dialog* or discuss or negotiat* or conversation*)) and health).mp. [mp=title, abstract, heading word, table of contents, key concepts, original title, tests & measures, mesh] | 33 |
| 5 | ((deliberative adj (dialog* or discuss or negotiat* or conversation*)) and health polic*).mp. | 2 |
| 6 | 2 or 3 or 4 | 351 |
| 7 | 1 and 6 | 17 |

**Global Health <1910 to 2021 Week 03>**

| **#** | **Search Statement** | **Results** |
| --- | --- | --- |
| 1 | africa*.mp. or exp Africa/ or (Algeria* or Angola* or Benin* or Botswana* or Burkina Faso or Burundi* or Cameroon* or Cape Verd* or Central African Republic or Chad or Tchad or Comoros or Congo or Congolese or Djibouti* or Egypt* or Equitorial Guinea or Eritrea* or Ethiopia* or Gabon or Gabonese or Gambia or Gambian or Ghana* or (Guinea not (fowl or pig*)) or Guinea Bissau or Ivory Coast or Kenya* or Lesotho or Liberia* or Libya* or Madagascar or Malawi* or Mali or Mauritania* or Mauritius or Morocco or Moroccan or Mozambique or Namibia* or Niger or Nigeria or Rwanda& or Sao Tome or Senegal* or Seychells or Sierra leone or Somalia* or Sough Africa* or Sudan* or Swaziland or Tanzania* or Togo or Tunisia* or Uganda* or Zambia* or Zimbabw*).ti,ab. | 347394 |
| 2 | ((policymaking or policy making).mp. or Policy Making/) and (dialog* or discuss* or negotiat* or conversation*).ti. | 21 |
| 3 | Health Policy/ and (dialog* or discuss* or negotiat* or conversation*).ti. | 124 |
| 4 | health polic*.mp. and (dialog* or discuss* or negotiat* or conversation*).ti. [mp=abstract, title, original title, broad terms, heading words, identifiers, cabicodes] | 139 |
| 5 | (((policy or policies) adj (dialog* or discuss or negotiat* or conversation*)) and health).mp. [mp=abstract, title, original title, broad terms, heading words, identifiers, cabicodes] | 133 |
| 6 | ((deliberative adj (dialog* or discuss or negotiat* or conversation*)) and health polic*).mp. | 13 |
| 7 | 2 or 3 or 4 or 5 | 257 |
| 8 | 1 and 7 | 75 |
| 9 | remove duplicates from 8 | 75 |

[**Cochrane Central Register of Controlled Trials**](https://www-cochranelibrary-com.login.ezproxy.library.ualberta.ca/) **Issue 1 of 12, January 2021
4 References**

4 Trials matching "#1 - ("policy dialog*" or "policy discuss*" or "policy negotiat*" or "deliberative dialog*" or "deliberative discuss*" or "deliberative negotiat*" ) and (africa* or Algeria* or Angola* or Benin* or Botswana* or Burkina Faso or Burundi* or Cameroon* or Cape Verd* or Central African Republic or Chad or Tchad or Comoros or Congo or Congolese or Djibouti* or Egypt* or Equitorial Guinea or Eritrea* or Ethiopia* or Gabon or Gabonese or Gambia or Gambian or Ghana* or (Guinea not (fowl or pig*)) or Guinea Bissau or Ivory Coast or Kenya* or Lesotho or Liberia* or Libya* or Madagascar or Malawi* or Mali or Mauritania* or Mauritius or Morocco or Moroccan or Mozambique or Namibia* or Niger or Nigeria or Rwanda or Sao Tome or Senegal* or Seychelles or Sierra Leone or Somalia* or South Africa* or Sudan* or Swaziland or Tanzania* or Togo or Tunisia* or Uganda* or Zambia* or Zimbabw*)"

**Cochrane Library**

Cochrane Database of Systematic Reviews : Issue 1 of 12, January 2021 2 references

There are 2 results from 10247 records for your search on '"policy dialog*" or "policy discuss*" or "policy negotiat*" or "deliberative dialog*" or "deliberative discuss*" or "deliberative negotiat*" and (africa* or Algeria* or Angola* or Benin* or Botswana* or Burkina Faso or Burundi* or Cameroon* or Cape Verd* or Central African Republic or Chad or Tchad or Comoros or Congo or Congolese or Djibouti* or Egypt* or Equitorial Guinea or Eritrea* or Ethiopia* or Gabon or Gabonese or Gambia or Gambian or Ghana* or (Guinea not (fowl or pig*)) or Guinea Bissau or Ivory Coast or Kenya* or Lesotho or Liberia* or Libya* or Madagascar or Malawi* or Mali or Mauritania* or Mauritius or Morocco or Moroccan or Mozambique or Namibia* or Niger or Nigeria or Rwanda or Sao Tome or Senegal* or Seychelles or Sierra Leone or Somalia* or South Africa* or Sudan* or Swaziland or Tanzania* or Togo or Tunisia* or Uganda* or Zambia* or Zimbabw*) in Cochrane Reviews'

**PROSPERO Searched January 24, 2021 Results =3**

Line Search for Hits

#1 "policy dialog*" or "policy discuss*" or "policy negotiat*" or

"deliberative dialog*" or "deliberative discuss*" or "deliberative negotiat*" 20

#2 africa* or Algeria* or Angola* or Benin* or Botswana* or "Burkina Faso"

or Burundi* or Cameroon* or "Cape Verd*" or "Central African Republic" or

Chad or Tchad or Comoros or Congo or Congolese or Djibouti* or Egypt* or

"Equitorial Guinea" or Eritrea* or Ethiopia* or Gabon or Gabonese or

Gambia or Gambian or Ghana* or (Guinea not (fowl or pig*)) or "Guinea

Bissau" or "Ivory Coast" or Kenya* or Lesotho or Liberia* or Libya* or

Madagascar or Malawi* or Mali or Mauritania* or Mauritius or Morocco

or Moroccan or Mozambique or Namibia* or Niger or Nigeria or Rwanda*

or "Sao" Tome or Senegal* or Seychelles or "Sierra Leone" or Somalia* or

"South Africa*" or Sudan* or Swaziland or Tanzania* or Togo or Tunisia* or

Uganda* or Zambia* or Zimbabw* 6228

#3 1 AND #2 3

**CINAHL Searched January 24, 2021**

| **#** | **Query** | **Results** |
| --- | --- | --- |
| S1 | (MH "Africa+") | 85,000 |
| S2 | Algeria* or Angola* or Benin* or Botswana* or "Burkina Faso" or Burundi* or Cameroon* or "Cape Verd*" or "Central African Republic" or Chad or Tchad or Comoros or Congo or Congolese or Djibouti* or Egypt* or "Equitorial Guinea" or Eritrea* or Ethiopia* or Gabon or Gabonese or Gambia or Gambian or Ghana* or (Guinea not (fowl or pig*)) or "Guinea Bissau" or "Ivory Coast" or Kenya* or Lesotho or Liberia* or Libya* or Madagascar or Malawi* or Mali or Mauritania* or Mauritius or Morocco or Moroccan or Mozambique or Namibia* or Niger or Nigeria or Rwanda* or "Sao" Tome or Senegal* or Seychelles or "Sierra Leone" or Somalia* or "South Africa*" or Sudan* or Swaziland or Tanzania* or Togo or Tunisia* or Uganda* or Zambia* or Zimbabw* | 91,826 |
| S3 | (MH "Policy Making") | 15,496 |
| S4 | "policy making" or policymaking | 17,633 |
| S5 | s3 or s4 | 17,633 |
| S6 | Ti (dialog* or discuss* or negotiat* or conversation*) | 23,926 |
| S7 | (MH "Health Policy+") | 88,962 |
| S8 | (S5 OR S7) | 101,965 |
| S9 | S6 AND S8 | 646 |
| S10 | (policy or policies) n1 (dialog* or discuss* or negotit* or conversation*) and health | 1,171 |
| S11 | ((deliberative N1 (dialog or discuss* or negotiat* or conversation*))) and (MH "Health Policy+" or "health polic*)) | 8 |
| S12 | (S9 OR S10 OR S11) | 1,764 |
| S13 | (S1 OR S2) | 103,030 |
| S14 | S12 AND S13 | 93 |

**Grey Literature Searches**

**BASE (Bielefeld Academic Search Engine) searched January 25, 2021**

tit:Policy tit:dialog* health* africa* doctype:(11* 12* 13 14 15 16 17 18* 19 F) = 34

**BASE (Bielefeld Academic Search Engine) searched January 25, 2021**

[individual country names substituted for multiple searches ] tit:policy tit:dialog* health doctype:1* = 45

**Google Scholar and Google Searched January 25, 2021**

africa health "policy dialog*" 61 items selected from first 10 pages
